# A MEDLINE categorization algorithm

**DOI:** 10.1186/1472-6947-6-7

**Published:** 2006-02-07

**Authors:** Stefan J Darmoni, Aurelie Névéol, Jean-Marie Renard, Jean-Francois Gehanno, Lina F Soualmia, Badisse Dahamna, Benoit Thirion

**Affiliations:** 1 CISMeF, Rouen University Hospital, 1, rue de Germont – 76031 Rouen, France; 2 Perception and Information Systems Laboratory & GCSIS, Medical School, University of Rouen, France; 3 CERIM, EA-2694, Medical School, University of Lille2, 1, Place de Verdun 59045 Lille Cedex, France

## Abstract

**Background:**

Categorization is designed to enhance resource description by organizing content description so as to enable the reader to grasp quickly and easily what are the main topics discussed in it. The objective of this work is to propose a categorization algorithm to classify a set of scientific articles indexed with the MeSH thesaurus, and in particular those of the MEDLINE bibliographic database. In a large bibliographic database such as MEDLINE, finding materials of particular interest to a specialty group, or relevant to a particular audience, can be difficult. The categorization refines the retrieval of indexed material. In the CISMeF terminology, metaterms can be considered as super-concepts. They were primarily conceived to improve recall in the CISMeF quality-controlled health gateway.

**Methods:**

The MEDLINE categorization algorithm (MCA) is based on semantic links existing between MeSH terms and metaterms on the one hand and between MeSH subheadings and metaterms on the other hand. These links are used to automatically infer a list of metaterms from any MeSH term/subheading indexing. Medical librarians manually select the semantic links.

**Results:**

The MEDLINE categorization algorithm lists the medical specialties relevant to a MEDLINE file by decreasing order of their importance. The MEDLINE categorization algorithm is available on a Web site. It can run on any MEDLINE file in a batch mode. As an example, the top 3 medical specialties for the set of 60 articles published in BioMed Central Medical Informatics & Decision Making, which are currently indexed in MEDLINE are: *information science*, *organization and administration *and *medical informatics*.

**Conclusion:**

We have presented a MEDLINE categorization algorithm in order to classify the medical specialties addressed in any MEDLINE file in the form of a ranked list of relevant specialties. The categorization method introduced in this paper is based on the manual indexing of resources with MeSH (terms/subheadings) pairs by NLM indexers. This algorithm may be used as a new bibliometric tool.

## Background

Categorization is designed to enhance resource description by organizing content description so as to enable the reader to grasp quickly and easily what a resource is about, what are the main topics discussed in it. In a previous study [1], we developed a categorization algorithm for health resources included in a quality-controlled health gateway called CISMeF (acronym of Catalogue and Index of Medical On-Line Resources in French) [2]. The relevance of the CISMeF Categorization Algorithm (CCA) was assessed on 123 randomly picked resources. The automatic categorization obtained was compared to the classified list of metaterms (or medical specialties) provided by a CISMeF librarian for each resource. The manual categorization was considered as the gold standard. This evaluation gave very satisfying results: 81% precision and 93% recall. For 63% of the resources, the automatic categorization was rated by the medical librarian as "fully relevant" or "fairly relevant" (whereas 20% of the resources were "partially relevant" and 22% of the resources were "non-relevant"). Therefore, in June 2004, the CISMeF team decided to implement this algorithm to generate resource categorization in the entire catalogue (N = 14,350 resources included in CISMeF -March 3, 2005-).

The objective of this paper is to propose a modified version of this categorization algorithm to classify a set of scientific articles indexed with the MeSH thesaurus and in particular those of the MEDLINE bibliographic database. Categorization allows a more general description with an upper level of granularity than MeSH indexing. In a large bibliographic database such as MEDLINE, finding materials of particular interest to a specialty group, or relevant to a particular audience, can be difficult. The categorization refines the retrieval of indexed material. This algorithm will be able to categorize the scientific production of one or several scientists or an entire research laboratory. It could also be used to categorize a set of articles of one peculiar journal. The categorization of articles from MEDLINE or scientific journals would characterize their contents by bringing out the medical specialties covered by each source.

## Methods

### CISMeF MeSH encapsulated terminology

The intent of the proposed categorization algorithm is to list the medical specialties relevant to a MEDLINE file including a set of articles by decreasing order of their importance. These medical specialties belong to the existing CISMeF terminology and they are called metaterms. The CISMeF terminology is based on the MeSH (Medical Subject Headings) thesaurus developed by the US National Library of Medicine [3].

The heterogeneity of Internet health resources led the CISMeF team to enhance the MeSH thesaurus with the introduction of two new concepts, respectively resource types (RT) and metaterms (MT), which have been previously described in [4].

**Figure 1:**
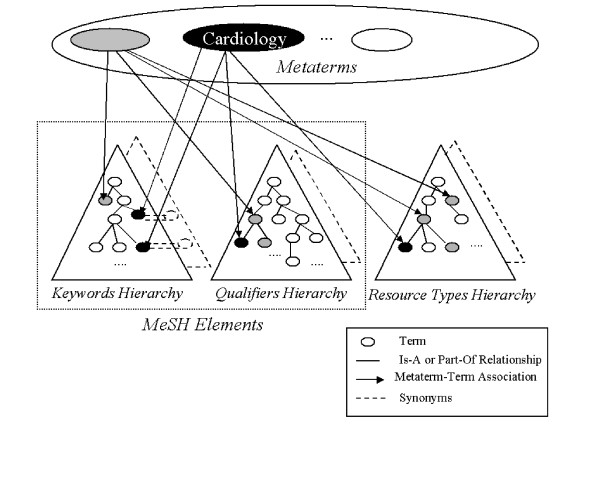
The CISMeF terminology structure.

A metaterm is generally a medical specialty or a biological science (e.g., cardiology or bacteriology) manually selected by the CISMeF chief librarian. For each metaterm (N = 115), semantic links were manually created with MeSH terms, MeSH subheadings and CISMeF resource types (N = 257), which are an extension of MEDLINE publication types (see Figure 1). These metaterms can be considered in the CISMeF terminology as super-concepts. A semantic link between a CISMeF term (MeSH terms, MeSH subheadings, or CISMeF resource types) and a CISMeF metaterm means that the CISMeF term is related to the concept denoted by the CISMeF metaterm. Therefore, an article indexed with the CISMeF term can be categorized by the corresponding metaterm: e.g. the MeSH term *psychiatric somatic therapies *is linked to the CISMeF metaterm *psychiatry *(see Table 1). There is a 0 to many relations between CISMeF terms and CISMeF metaterms: e.g. the MeSH term 'Paris' has no semantic link with any of the CISMeF metaterms. On the contrary, the MeSH term *acquired immunodeficiency syndrome *is linked to two different metaterms: '*virology*' and *allergy and immunology*. The semantic links were created on the basis of the CISMeF librarians' technical know-how and the expertise of medical specialists from the Rouen University Hospital, France.

**Table 1:** Semantic links of the MT psychiatry

"community mental health centers"[MeSH Terms]
"community mental health centers"[CISMeF Resource Type]
"diagnostic and statistical manual of mental disorders"[MeSH Terms]
"hospitals, psychiatric"[MeSH Terms]
"hospitals, psychiatric"[CISMeF Resource Type]
"mental disorders"[MeSH Terms]
"mentally Ill persons"[MeSH Terms]
"psychiatric department, hospital"[MeSH Terms]
"psychiatric department, hospital"[CISMeF Resource Type]
"psychiatric somatic therapies"[MeSH Terms]
"psychiatry"[MeSH Terms]
"psychophysiologic disorders"[MeSH Terms]
"psychotherapy"[MeSH Terms]
"psychotropic drugs"[MeSH Terms]
"schizophrenic psychology"[MeSH Terms]

As defined by the DC Metadata Initiative [6], a RT is used to categorize the nature of the content of the resource. MeSH (term/subheading) pairs describe the topic of the resource. Internet health resources being more heterogeneous than MEDLINE scientific articles, the CISMeF RTs are more diverse than the publication types (PT) of MEDLINE. Specific RTs are dedicated to electronic health resources, such as *association*, *patient information*, *community networks*, or *clinical guidelines*. For example, in the case of a clinical guideline about carbon monoxide intoxication, *carbon monoxide poisoning *is the MeSH term and *clinical guidelines *is the resource type. CISMeF RTs are organized similarly to MeSH terms and subheadings, in a hierarchical structure with subsumption relationships (allowing the explode property) and a maximum of five-level depth. The Medline publication types are mainly a flat list (see [7]). The controlled list of RTs is available at [8]. The RT list has been manually built and maintained by the CISMeF team since 1997. Nonetheless, this list is largely driven from the MeSH thesaurus as 187 RTs (76%) are also MeSH terms (e.g. *magnetic resonance imaging*) and 28 RTs (11%) are also MEDLINE publication types (e.g. *technical report*).

These medical specialties are in most cases also MeSH terms, in the G02.403 MeSH tree. For example, the metaterm *psychiatry *is linked to the MeSH terms *psychiatry *(and all the MeSH terms below in the tree structure) and *psychiatric hospital *that belong to a completely different tree structures within the MeSH and also with the CISMeF resource type *mental health dispensary *(see Table 1: Semantic Links of the metaterm Psychiatry). The list of metaterms are available at [9]. Clicking on a metaterm will launch a complex query on the set of MeSH terms and subheadings linked to the metaterm.

In 1997, the primary use of metaterms was to address the relatively restrictive nature of some MeSH terms in information retrieval [10]. The main objective was to improve recall by using metaterms instead of MeSH terms – therefore expanding the queries submitted to the CISMeF health gateway. To illustrate the difference between MeSH terms and metaterms in terms of information retrieval in the CISMeF health gateway, let us submit the two following sample queries to the Boolean Search of the Doc'CISMeF search engine [11]: *'guidelines in cardiology' *or *'databases in virology'*, where '*guidelines' *and *'databases' *are CISMeF resource types and '*cardiology' *and '*virology' *are viewed alternatively as MeSH terms and CISMeF metaterms. The query *'guidelines in cardiology' *retrieves 11 resources when *'cardiology' *is considered as a MeSH term (Boolean query: '*guidelines.tr AND cardiology.mc'*, where tr stands for resource type and mc stands for MeSH term) vs. 143 resources when *'cardiology' *is considered as a MT (Boolean query: '*guidelines.tr AND cardiology.mt'*, where mt stands for metaterm). The query *'databases in virology' *retrieves 0 resource when *virology *is considered as a MeSH term (Boolean query: *databases.tr AND virology.mc) *vs. 4 resources when *virology *is considered as a MT (Boolean query: *databases.tr AND virology.mt*).

### Categorization algorithm

The MEDLINE categorization algorithm (MCA) is based on the CISMeF librarians' technical know-how. The (CISMeF) semantic links existing between MeSH terms and metaterms on the one hand and between subheadings and metaterms on the other hand are used to automatically infer a list of metaterms (medical specialties) from any MeSH term/subheading indexing. The categories of the MEDLINE categorization algorithm are the metaterms. As an example, because the CISMeF librarians have created a semantic link between the MeSH term *psychotherapy *and the metaterm *psychiatry*, *psychiatry *will automatically be inferred for every MEDLINE article indexed with ps*ychotherapy*. Hence, a metaterm categorization can be automatically inferred for each MEDLINE article manually indexed by NLM indexers. This process is performed recursively to obtain the list of metaterms related to any MEDLINE file obtained from any MEDLINE query.

If a MeSH term is semantically linked to several metaterms, more than one metaterm will be inferred. For example, the term *thumb *induces the metaterm *anatomy*, and the term *alcoholism *induces both the metaterms *psychiatry *and *toxicology*. In this case, the MTs are not weighed differently. Similarly, the MeSH term/subheading pair *alcoholism / legislation & jurisprudence *induces the metaterms *psychiatry *(from the semantic link between *alcoholism *and *psychiatry*), *toxicology *(from the semantic link between *alcoholism *and *toxicology*) and *medical law *(from the semantic link between *legislation & jurisprudence *and *medical law*).

Assume there are in a set of MEDLINE articles to be categorized:

n MeSH terms T_1_, T_2_, ..., T_n _(major terms are marked by a star),

m subheadings Q_1_, Q_2_, ..., Q_m _(major subheadings coming from major pairs term/subheading).

The CISMeF terminology enables us to infer k metaterms M_1_, M_2_, ..., M_k _from these sets of terms. For each metaterm M_i_, a *major score *and a *minor score *are computed using the formulas (1) and (2). We define the major score as being the number of *major *(or starred) indexing terms and subheadings from which the metaterm M_i _is inferred. The minor score is the number of *minor *indexing terms and subheadings from which the metaterm M_i. _is inferred.

*major*(M_i_) = Card {T_i_*/T_i_*implies M_i_} + Card{Q_i_*/Q_i_* implies M_i_}     (1)

*minor*(M_i_) = Card{ T_i _/ T_i _implies M_i_} + Card{Q_i_/ Q_i _implies M_i_}     (2)

Subsequently, metaterms are classified by decreasing major scores, and in the case of similar major scores, minor scores are used in order to obtain the final semantic categorization. The number of metaterms used to classify one article may vary. It depends on the number of MeSH terms (and MeSH terms/subheading pairs) assigned to the article and the number of semantic links between MeSH terms, Subheadings and Metaterms.

There are two main differences between the MEDLINE categorization algorithm and the previous CISMeF categorization algorithm [1]. One concerns the method and one concerns the scope of the categorization:

• Method: The MEDLINE categorization algorithm does not take into account the semantic links between CISMeF metaterms and CISMeF resources types because it will have to restrain to the few resources types which are also MEDLINE publication types (N = 28 out of 257, 11%). Using such semantic links would have introduced a major bias. Therefore, we decided not to use them in the MEDLINE categorization algorithm. Furthermore, this strategic choice was driven by the fact that MEDLINE publication types are much less suitable to categorize medical specialties when compared to CISMeF resources types (e.g. CISMeF RT *lecture *notes for CISMeF MT *medical education *or CISMeF RT *echography *for CISMeF MT *medical imaging*). Finally, only the following six metaterms could have been linked to the CISMeF resources types which are also MEDLINE publication types (N = 9 out of 28):

• the CISMeF MT *statistics *with CISMeF RT (and MEDLINE PT) *meta-analysis*,

• the CISMeF MT *information science *with CISMeF RT (and MEDLINE PT) *database*,

• the CISMeF MT *Evidence-Based Medicine *with CISMeF RT (and MEDLINE PT) *consensus development conferences*,

• the CISMeF MT *Medical Law *with CISMeF RT (and MEDLINE PT) *Legislation*,

• the CISMeF MT *medical education *with CISMeF RTs (and MEDLINE PTs) *examination questions, instruction motion picture & problems and exercises*,

• the CISMeF MT *patient *with CISMeF RTs (and MEDLINE PTs) *patient education handout *&*popular works*.

The other 19 shared CISMeF resources types / MEDLINE publication types (e.g. *portrait *or *table*) have no semantic link with any of CISMeF metaterm.

• Scope: The CISMeF categorization algorithm was used on only one CISMeF resource description whereas the MEDLINE categorization algorithm is intended to be more global and may be applied to a collection of citations (MEDLINE file). In that case, it is recursively applied to each citation of the collection. It is also possible to obtain a MEDLINE categorization for only one citation.

### Creation of the MEDLINE Categorization Web site

**Figure 2:**
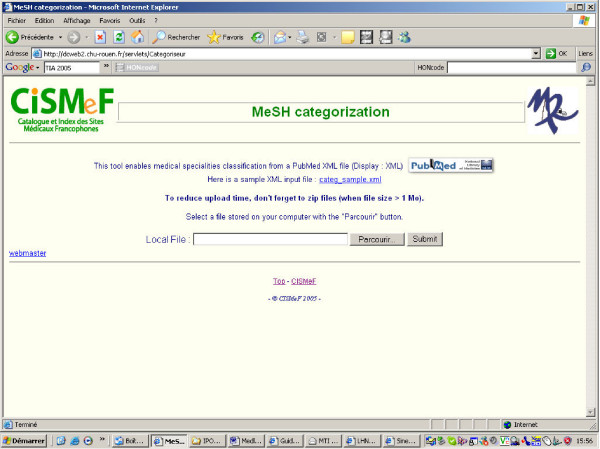
the MEDLINE Categorization Web site's Home Page.

First, users need to log into the MEDLINE Categorization Web site (see Figure 2). Then, they have to perform their query on the MEDLINE bibliographic database, using the PubMed Web site [12] and to save it on a file using the XML format. The MEDLINE Categorization Website will upload the MEDLINE file obtained as described as an input and compute the relevant categories. The CISMeF team developed an XML parser to extract MeSH terms and subheadings and their respective Major/Minor indexing. A servlet written in Java implements the MEDLINE categorization algorithm, and a Java Bean dispatches the query result towards a Java Server Page (JSP). This Java code relies on a xerces SAX Parser for XML parsing, and an Oracle OCI connection to our database, which contains the CISMeF terminology (including MeSH terms, subheadings and RT hierarchies and the semantic links between the CISMeF MTs and the rest of the terminology). This program was primarily designed to be processed in a batch mode. Nonetheless, if the MEDLINE file is zipped before submission (this step is warmly recommended on the Web site), the processing time is acceptable: between 5 to 10 seconds for 1.000 articles. It is linearly proportional to the number of articles submitted to the algorithm.

## Results

The access to the MEDLINE categorization website is currently restricted to the Rouen University Hospital Extranet (ID/password needed) [13], but will be soon freely available on the Internet.

**Figure 3:**
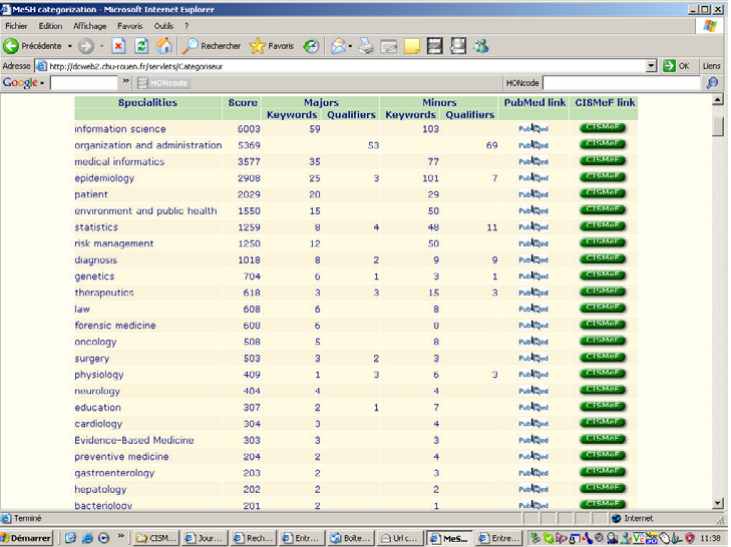
The MEDLINE Categorization algorithm results for the BMC medical informatics and decision making Journal.

An example of the MEDLINE categorization algorithm is displayed in Figure 3 for the 60 articles published in this journal BioMed Central (BMC) Medical Informatics & Decision Making, which are currently indexed in MEDLINE (March 27, 2005). The top 3 medical specialties for this set of articles are: 'information science', 'organization and administration' and 'medical informatics'.

## Discussion

The MEDLINE categorization algorithm may have several uses in bibliometrics:

• To categorize the scientific production of one scientist (or a group of scientists or an entire laboratory): for example, to check if its main fields of research are correlated with the top-rated metaterms generated by the algorithm.

• To categorize the articles of one journal to check if the main fields of coverage are correlated with the top-rated metaterms generated by the algorithm, as shown in Figure 3. Not surprisingly, among the Top 10 medical specialties calculated by the MCA for the journal BMC Medical Informatics & Decision Making, we found *medical informatics *(ranked second), *information science *(ranked first, but since it is located (L01) above *medical informatics *(L01.700) in the MeSH L tree it is inferred by the algorithm at least as many times as *medical informatics*, *epidemiology *(ranked fourth, with a score very close to *medical informatics*) and *statistics *(ranked sixth, and which is also very close to *medical informatics*). The other Top 10 medical specialties (*organization & administration, patient, environment and public health, risk management, diagnosis, genetics and therapeutics*) indicate the main trends of the BMC Medical Informatics & Medical Decision Making Journal and therefore have some interest for the editorial board of this journal, for scientists likely to publish in this journal and also for readers of this electronic journal freely available on the Internet.

As mentioned by one of the reviewers (SN), the indexing of citations is aimed at indicating topics discussed, not at indicating the persons or specialty to whom the citation might be of interest. This information model has the advantage of not requiring that all possible views of why a citation might be of interest be recognized before indexing. The disadvantage is that a search for information of interest in broad categories is not well supported. The use in MeSH of a specialty name as an index term implies that the article cited is about the specialty, not that the article would be of interest to that specialty. For MEDLINE, MeSH is used to index the most specific aspects of citations. In contrast, some other indexing systems intend to capture the broadest aspects of a citation, or possibly even the disciplines involved.

No formal evaluation of the MEDLINE categorization algorithm presented was performed. However, an indication on performance may be provided by the formal evaluation of the CISMeF categorization algorithm, which is quite similar.

The validity of the semantic links between MEDLINE terms & subheadings and CISMeF metaterms may be questioned, and (moreover) the sole contribution of CISMeF experts to their creation may constitute a considerable bias. Since the CISMeF categorization algorithm was primarily developed, several improvements have been implemented: firstly with the help of several medical experts from the Rouen University Hospital; secondly and mostly, with the help of the Network of National Library of Medicine (NNLM; [14]), using the Medlib-L listserv [15]. Several medical librarians of the NNLM proposed some improvements to the MTs semantic links: mostly, they helped to reduce the false negative results, when proposing new semantic links with MeSH terms and subheadings.

Although there is no similar tool available to our knowledge, Bodenreider described a similar categorization algorithm based on UMLS semantics and MeSH disease categories (N = 22) [5]. According to [4], the UMLS algorithm performs better than ours (relevance of 92% vs. precision and recall of 81% and 93%). However, the MEDLINE categorization algorithm is able to classify scientific articles among 115 different specialties whereas the Bodenreider's algorithm works with 22 MeSH disease categories. Furthermore, the CISMeF MTs are broader than the MeSH disease categories, which are all included in a single CISMeF MT. By combining the two methods, we could extend the categorization possibilities to medical resources indexed with other UMLS terminologies, such as the ICD 10 for clinical reports. In fact, the UMLS provides links between more than 70 terminologies including the MeSH to UMLS concepts. Indeed, it is possible to map these terminologies to the MeSH, and thus to the CISMeF metaterms in order to obtain the categorization.

CISMeF MTs can be viewed as quite similar to JDs (Journal Descriptors) used for indexing journals *per se *(see [16]; List of journals indexed for Medline, 2005). Humphrey has developed the JDI (Journal Descriptor Indexing) system based on the statistical associations between JDs and text words or starred (major) MeSH terms [17]. In the near future, we will compare the respective precision of the human-driven metaterm algorithm *vs*. the machine-driven journal descriptor algorithm on a sample of MEDLINE articles. After this study, we will collaborate with Humphrey to improve semantic links between MTs and MeSH terms, using statistical associations between JDs and MeSH terms. We also plan to use statistical associations between JDs and MeSH terms to improve information retrieval in the CISMeF search engine, limiting the scope of a query by proposing the most frequently associated JDs (e.g. for the query 'asthma', the system will suggest restricting its scope to the JDs 'Critical care' or 'Pediatrics').

CISMeF MTs can also be viewed as quite similar to PubMed Subsets in the context of information retrieval. They are both devoted to optimize information retrieval but have different goals: search filtering [18] limiting the number of articles to read in case of the PubMed Subsets. In most cases, expanding the request to maximize the recall for the CISMeF MT (e.g. guidelines in cardiology) [4]. Nonetheless, CISMeF medical librarians use also CISMeF MTs for search filtering purposes (e.g. the query wood dust exposure in occupational medicine would be transformed manually to the following CISMeF query 'wood dust exposure [MeSH term] AND occupational medicine [Metaterm]'). The second difference is the respective number of PubMed Subsets vs. CISMeF MTs (respectively 7 vs. 115). Finally, there is potentially more noise with the PubMed Subsets when compared with CISMeF MTs: PubMed Subsets are pre-built queries using keywords, text words and finally MEDLINE (or PubMed) journal titles. CISMeF MTs may only be semantically linked to MeSH terms, subheadings and CISMeF RTs. Nonetheless, PubMed Subsets may be more optimal in terms of recall, because of the "PubMed in process" citations, which are not yet manually indexed with MeSH terms, subheadings and publication types.

In the near future, the MEDLINE Categorization algorithm will be integrated in the SIGAPS project [19], which is directed by the Department of Research and the Department of Medical Informatics (CERIM) of the University Hospital of Lille – France. SIGAPS aims to develop a bibliographic full-Web application designed for scientific publications analysis. A functional evaluation of SIGAPS is being carried out at 8 University Hospitals throughout France. SIGAPS evaluates each MEDLINE citation on a six-level scale. Then, SIGAPS generates aggregated data from the hierarchical structure of a hospital or other institution, and enables analysis and visualization of the results on a Web browser. The integration of the Medline Categorization Algorithm will improve the capacity of the tool to compute statistics related to the different interests and trends of unique researchers or labs.

## Conclusion

We have presented a MEDLINE categorization algorithm designed by the CISMeF team in order to classify the medical specialties addressed in any MEDLINE file in the form of a ranked list of relevant specialties. The categorization method introduced in this paper is based on the manual indexing of resources with MeSH (terms/subheadings) pairs by NLM indexers. This algorithm may be used as a new bibliometric tool.

## Competing interests

The author(s) declare that they have no competing interests.

## Authors' contributions

SJ. Darmoni, A. Neveol, LF. Soualmia and B. Thirion have created the CISMeF terminology and has conceptualized the MEDLINE categorization algorithm. B. Dahamna has implemented the MCA in the CISMeF Web site. JM. Renard & JF. Gehanno have tested the MCA in its different versions. JM. Renard is integrating the MCA in the SIGAPS environment. All the authors have participating in the writing of the manuscript.

## Pre-publication history

The pre-publication history for this paper can be accessed here:


